# Modifiable Risk Factors for Alzheimer Disease and Related Dementias Among Adults Aged ≥45 Years — United States, 2019

**DOI:** 10.15585/mmwr.mm7120a2

**Published:** 2022-05-20

**Authors:** John D. Omura, Lisa C. McGuire, Roshni Patel, Matthew Baumgart, Raza Lamb, Eva M. Jeffers, Benjamin S. Olivari, Janet B. Croft, Craig W. Thomas, Karen Hacker

**Affiliations:** ^1^National Center for Chronic Disease Prevention and Health Promotion, CDC; ^2^CyberData Technologies, Inc., Herndon, Virginia; ^3^Alzheimer’s Association, Chicago, Illinois; ^4^Oak Ridge Institute for Science and Education, Oak Ridge, Tennessee.

Alzheimer disease,* the most common cause of dementia, affects an estimated 6.5 million persons aged ≥65 years in the United States (*1*). A growing body of evidence has identified potential modifiable risk factors for Alzheimer disease and related dementias (ADRD) (*1*–*3*). In 2021, the National Plan to Address Alzheimer's Disease (National Plan) introduced a new goal to “accelerate action to promote healthy aging and reduce risk factors for Alzheimer’s disease and related dementias” to help delay onset or slow the progression of ADRD (*3*). To assess the status of eight potential modifiable risk factors (i.e., high blood pressure, not meeting the aerobic physical activity guideline, obesity, diabetes, depression, current cigarette smoking, hearing loss, and binge drinking), investigators analyzed data from the cognitive decline module that was administered to adults aged ≥45 years in 31 states and the District of Columbia (DC)^†^ in the 2019 Behavioral Risk Factor Surveillance System (BRFSS) survey. Among the risk factors, prevalence was highest for high blood pressure (49.9%) and lowest for binge drinking (10.3%) and varied by selected demographic characteristics. Adults with subjective cognitive decline (SCD),^§^ an early indicator of possible future ADRD (*4*), were more likely to report four or more risk factors than were those without SCD (34.3% versus 13.1%). Prevalence of SCD was 11.3% overall and increased from 3.9% among adults with no risk factors to 25.0% among those with four or more risk factors. Implementing evidence-based strategies to address modifiable risk factors can help achieve the National Plan’s new goal to reduce risk for ADRD while promoting health aging.^¶^^,^**

BRFSS is a cross-sectional, random-digit–dialed, annual telephone survey of noninstitutionalized U.S. adults aged ≥18 years. BRFSS is administered by state and territorial health departments, and responses are weighted to produce data representative of each state. The 2019 combined (landline and mobile) median response rate was 49.4%.^††^ In 2019, the cognitive decline module was administered to adults aged ≥45 years in 31 states and DC.

Eight modifiable risk factors were assessed: high blood pressure, not meeting the aerobic physical activity guideline, obesity, diabetes, depression, current cigarette smoking, hearing loss, and binge drinking.^§§^ The total number of risk factors per respondent was defined as the sum of any risk factors reported and was grouped into no, one, two, three, or four or more risk factors. Respondents were classified as experiencing SCD if they responded “yes” when asked if they had experienced worsening or more frequent confusion or memory loss in the previous 12 months. Data were collected from 161,941 respondents; 21,865 (13.5%) respondents who refused to respond to the question assessing SCD or who responded, “don’t know/not sure,” were excluded. Respondents with missing data for risk factors (ranging from 0.2% for diabetes to 8.8% for obesity) were excluded from corresponding prevalence estimate calculations.

Prevalence of each modifiable risk factor was estimated overall and by SCD status and selected demographic characteristics. The proportion of respondents with no, one, two, three, or four or more risk factors was determined by SCD status. Prevalence of SCD was determined among respondents with and without each risk factor and by number of risk factors. All percentages were weighted and unadjusted. Analyses were conducted using SAS-callable SUDAAN (version 9.4; SAS Institute) to account for complex survey design and weighting. T-tests were used to determine statistically significant differences by subgroup (p<0.05). All estimates met reliability standards by having a relative SE <30%. This activity was reviewed by CDC and was conducted consistent with applicable federal law and CDC policy.^¶¶^

In 2019, the prevalence of SCD among adults aged ≥45 years in 31 participating states and DC was 11.3% (Table 1). The most common modifiable risk factor for ADRD was high blood pressure (49.9%), followed by not meeting the aerobic physical activity guideline (49.7%), obesity (35.3%), diabetes (18.6%), depression (18.0%), current cigarette smoking (14.9%), hearing loss (10.5%), and binge drinking (10.3%). The prevalences of risk factors varied by selected demographic characteristics, including race and ethnicity. For example, the prevalence of several risk factors was higher among adults who were American Indian or Alaska Native, non-Hispanic Black or African American, or Hispanic, than among non-Hispanic White adults. Adults with SCD were more likely to report most of the modifiable risk factors and were more likely to report four or more risk factors (34.3%) than were those without SCD (13.1%) (Figure).

**TABLE 1:** Prevalence of selected modifiable risk factors* among adults aged ≥45 years, by selected characteristics and subjective cognitive decline^†^ status — Behavioral Risk Factor Surveillance System, United States,^§^ 2019

Characteristic	Sample	Prevalence of risk factors, % (95% CI)							
	No. (%)^¶^	High blood pressure	Not meeting aerobic physical activity guideline	Obesity	Diabetes	Depression	Current cigarette smoking	Hearing loss	Binge drinking
**Overall**	**140,076**	**49.9 (49.4–50.4)**	**49.7 (49.2–50.3)**	**35.3 (34.8–35.8)**	**18.6 (18.2–19.0)**	**18.0 (17.6–18.4)**	**14.9 (14.6–15.3)**	**10.5 (10.2–10.8)**	**10.3 (9.9–10.6)**
**Age group, yrs**									
45–54**	27,500 (28.3)	36.3 (35.3–37.4)	52.7 (51.6–53.9)	39.0 (37.9–40.1)	12.5 (11.7–13.2)	19.9 (19.1–20.8)	18.6 (17.8–19.5)	4.8 (4.4–5.3)	16.2 (15.4–17.0)
55–64	39,421 (30.9)	47.8 (46.8–48.8)**^††^**	50.3 (49.3–51.4)**^††^**	37.9 (37.0–38.9)	17.9 (17.1–18.6)**^††^**	20.7 (19.9–21.4)**^††^**	18.8 (18.0–19.5)	7.9 (7.3–8.5)**^††^**	11.9 (11.2–12.6)**^††^**
65–74	42,016 (24.2)	59.0 (58.0–59.9)**^††^**	46.2 (45.2–47.1)**^††^**	34.9 (34.0–35.8)**^††^**	23.8 (22.9–24.6)**^††^**	17.2 (16.5–17.9)**^††^**	12.3 (11.7–13.0)**^††^**	12.7 (12.1–13.4)**^††^**	6.6 (6.0–7.1)**^††^**
>75	31,139 (16.6)	63.7 (62.6–64.8)**^††^**	48.6 (47.5–50.0)**^††^**	24.7 (23.7–25.6)**^††^**	23.0 (22.0–23.9)**^††^**	11.2 (10.5–11.9)**^††^**	5.4 (4.9–5.9)**^††^**	22.0 (21.1–22.9)**^††^**	2.7 (2.3–3.1)**^††^**
**Sex**									
Men**	60,436 (46.6)	52.5 (51.7–53.3)	46.9 (46.1–47.7)	35.6 (34.8–36.3)	20.1 (19.5–20.7)	12.9 (12.4–13.5)	15.9 (15.3–16.5)	13.3 (12.8–13.9)	14.0 (13.4–14.6)
Women	79,640 (53.4)	47.6 (46.9–48.3)**^††^**	52.2 (51.5–52.9)**^††^**	35.0 (34.3–35.7)	17.3 (16.8–17.8)**^††^**	22.5 (21.9–23.1)**^††^**	14.1 (13.6–14.6)**^††^**	8.1 (7.7–8.4)**^††^**	7.1 (6.7–7.4)**^††^**
**Sexual and gender minority status**									
Non-LGBT**	87,585 (96.1)	50.2 (49.5–50.8)	48.5 (47.8–49.2)	35.0 (34.3–35.6)	18.5 (18.0–19.0)	17.6 (17.1–18.1)	14.7 (14.2–15.2)	10.2 (9.8–10.6)	10.4 (9.9–10.8)
LGBT	3,226 (3.9)	49.2 (45.6–52.7)	53.9 (50.3–57.5)**^††^**	33.7 (30.4–37.0)	20.5 (17.9–23.0)	25.4 (22.4–28.5)**^††^**	18.1 (15.5–20.7)**^††^**	10.1 (8.5–11.7)	13.4 (10.9–15.9**^††^**
**Race/Ethnicity**									
American Indian or Alaska Native, non-Hispanic	2,059 (1.2)	54.1 (48.7–59.5)**^††^**	59.8 (55.1–64.6)**^††^**	39.4 (34.4–44.4)	24.7 (20.7–28.7)**^††^**	22.9 (18.0–27.8)	26.5 (21.8–31.1)**^††^**	17.5 (12.9–22.1)**^††^**	9.6 (6.8–12.4)
Asian or Pacific Islander, non-Hispanic	923 (2.0)	35.8 (29.8–41.8)**^††^**	45.6 (39.2–52.0)	13.6 (9.8–17.5)**^††^**	19.0 (14.1–23.9)	7.2 (3.2–11.2)**^††^**	5.4 (3.0–7.9)**^††^**	4.4 (2.0–6.8)**^††^**	5.0 (2.4–7.6)**^††^**
Black, non-Hispanic	11,947 (12.4)	64.7 (63.0–66.4)**^††^**	57.8 (56.1–59.6)**^††^**	45.0 (43.2–46.7)**^††^**	27.2 (25.7–28.7)**^††^**	15.7 (14.4–17.0)**^††^**	17.5 (16.1–18.8)**^††^**	6.4 (5.6–7.1)**^††^**	8.2 (7.3–9.1)**^††^**
Hispanic	5,927 (9.1)	44.3 (41.5–47.0)**^††^**	58.6 (55.7–61.4)**^††^**	37.3 (34.5–40.1)**^††^**	23.3 (21.1–25.6)**^††^**	15.9 (14.0–17.9)**^††^**	13.1 (11.2–15.0)	8.6 (6.9–10.3)**^††^**	11.6 (9.5–13.8)
White, non-Hispanic**	113,697 (73.8)	48.5 (48.0–49.0)	47.2 (46.6–47.7)	33.9 (33.4–34.4)	16.5 (16.1–16.8)	18.9 (18.4–19.3)	14.7 (14.4–15.1)	11.3 (11.0–11.7)	10.7 (10.3–11.0)
Multiple races, non-Hispanic	1,848 (1.0)	52.3 (48.3–56.2)	50.5 (46.4–54.6)	39.3 (35.4–43.2)**^††^**	22.5 (19.3–25.8)**^††^**	23.9 (20.8–26.9)**^††^**	25.1 (21.6–28.5)**^††^**	13.5 (10.9–16.2)	11.6 (8.6–14.6)
Other race, non-Hispanic**^§§^**	920 (0.6)	46.5 (40.7–52.3)	52.6 (46.4–58.7)	34.7 (28.8–40.5)	20.1 (15.2–25.1)	20.1 (15.1–25.1)	15.2 (11.0–19.3)	12.5 (9.1–15.9)	9.8 (6.4–13.1)
**Education level**									
Not a high school graduate**^¶¶^	10,172 (12.7)	58.1 (56.1–60.1)	67.1 (65.1–69.0)	37.0 (35.1–38.9)	26.9 (25.1–28.6)	23.3 (21.7–25.0)	25.9 (24.3–27.6)	15.4 (14.0–16.8)	9.8 (8.4–11.3)
High school graduate	38,766 (28.8)	54.4 (53.4–55.4)**^††^**	56.6 (55.6–57.6)**^††^**	38.4 (37.4–39.3)	20.5 (19.8–21.3)**^††^**	18.0 (17.3–18.8)**^††^**	19.0 (18.3–19.8)**^††^**	12.2 (11.6–12.8)**^††^**	10.5 (9.9–11.1)
Some college or more	90,690 (58.5)	46.0 (45.4–46.6)**^††^**	42.5 (41.9–43.2)**^††^**	33.4 (32.8–34.0)**^††^**	15.9 (15.5–16.4)**^††^**	16.9 (16.5–17.4)**^††^**	10.6 (10.2–11.0)**^††^**	8.7 (8.4–9.0)**^††^**	10.3 (9.9–10.7)
**Employment status**									
Employed**^,^***	55,023 (45.1)	39.1 (38.3–39.9)	47.6 (46.7–48.4)	35.9 (35.1–36.7)	11.9 (11.4–12.4)	12.7 (12.2–13.2)	13.6 (13.1–14.2)	5.7 (5.3–6.0)	14.7 (14.1–15.3)
Unemployed	3,987 (3.8)	49.0 (45.5–52.5)**^††^**	52.2 (48.5–55.9)**^††^**	36.3 (33.1–39.5)	18.2 (16.0–20.5)**^††^**	29.5(26.6–32.5)**^††^**	28.6 (25.6–31.6)**^††^**	9.5 (6.4–12.5)**^††^**	15.4 (11.9–19.0)
Retired	61,309 (35.7)	60.3 (59.5–61.1)**^††^**	44.5 (43.7–45.3)**^††^**	31.9 (31.2–32.7)**^††^**	22.8 (22.1–23.5)**^††^**	14.9 (14.3–15.4)**^††^**	10.5 (10.0–11.0)**^††^**	15.7 (15.1–16.2)**^††^**	5.9 (5.5–6.3)**^††^**
Unable to work	12,686 (10.5)	64.8 (63.1–66.4)**^††^**	74.9 (73.4–76.3)**^††^**	46.0 (44.3–47.8)**^††^**	34.6 (33.0–36.3)**^††^**	48.0 (46.3–49.7)**^††^**	32.9 (31.3–34.5)**^††^**	16.2 (15.0–17.3)**^††^**	7.2 (6.4–8.0)**^††^**
Other^†††^	6,251 (4.9)	43.4 (40.9–45.9)**^††^**	51.1 (48.4–53.7)**^††^**	30.7 (28.3–33.0)**^††^**	15.8 (13.9–17.6)**^††^**	18.3 (16.3–20.2)**^††^**	11.1 (9.4–12.7)**^††^**	6.5 (5.5–7.5)	4.6 (3.8–5.5)**^††^**
**Has a primary care provider**									
Yes**	125,402 (88.5)	52.3 (51.7–52.8)	49.1 (48.5–49.6)	36.0 (35.5–36.6)	19.9 (19.4–20.3)	18.5 (18.1–19.0)	13.6 (13.3–14.0)	10.7 (10.4–11.1)	9.6 (9.3–10.0)
No	14,155 (11.5)	31.9 (30.2–33.5)**^††^**	54.5 (52.6–56.3)**^††^**	29.4 (27.8–31.0)**^††^**	9.0 (8.0–9.9)**^††^**	14.3 (13.2–15.5)**^††^**	24.8 (23.4–26.3)**^††^**	8.7 (7.5–9.9)**^††^**	15.4 (14.0–16.8)**^††^**
**SCD^†^**									
Yes	15,608 (11.3)	60.9 (59.4–62.4)**^††^**	63.5 (62.0–64.9)**^††^**	39.2 (37.7–40.7)**^††^**	28.7 (27.3–30.2)**^††^**	45.6 (44.1–47.2)**^††^**	24.4 (23.0–25.8)**^††^**	23.1 (21.9–24.3)**^††^**	10.3 (9.4–11.4)
No**	124,468 (88.7)	48.5 (47.9–49.1)	48.0 (47.4–48.5)	34.8 (34.2–35.3)	17.3 (16.9–17.8)	14.5 (14.2–14.9)	13.8 (13.4–14.1)	8.9 (8.6–9.2)	10.3 (9.9–10.7)

**Abbreviations:** LGBT = lesbian, gay, bisexual, or transgender; SCD = subjective cognitive decline.

* Behavioral Risk Factor Surveillance System survey questions and calculated variables for 2019 are available at https://www.cdc.gov/brfss/questionnaires/pdf-ques/2019-BRFSS-Questionnaire-508.pdf and https://www.cdc.gov/brfss/annual_data/2019/pdf/2019-calculated-variables-version4-508.pdf, respectively. Not meeting the aerobic physical activity guideline was defined as answering “no” to question C11.01 or reporting <150 minutes per week of moderate-intensity aerobic activity, or <75 minutes per week of vigorous-intensity aerobic activity, or an equivalent combination of the two based on questions C11.02–C11.07 consistent with the Physical Activity Guidelines for Americans, 2nd edition (https://health.gov/our-work/nutrition-physical-activity/physical-activity-guidelines/current-guidelines). Obesity was defined as having a calculated body mass index of ≥30 kg/m^2^ based on self-reported height and weight from questions C08.17 and C08.18. High blood pressure, diabetes, depression, and hearing loss were defined as answering “yes” to questions C04.01 (excluding pregnancy-related high blood pressure), C06.11 (excluding pregnancy-related diabetes), C06.09, and C08.20, respectively. Binge drinking was defined as reporting having had one or more alcoholic beverages in the previous 30 days and responding “one or more” when asked how many times during the past 30 days they had had X [X = 5 for men and X = 4 for women] or more drinks on an occasion (questions C10.01 and C10.03, respectively). Current cigarette smoking was defined as reporting having smoked ≥100 cigarettes in their lifetime and now smoking every day or some days in response to questions C09.01 and C09.02, respectively.

^†^ SCD was defined as the self-reported experience of worsening confusion or memory loss in the previous year.

^§^ The following U.S. jurisdictions administered the SCD module in 2019: Alabama, Connecticut, District of Columbia, Florida, Georgia, Indiana, Iowa, Kansas, Louisiana, Maryland, Michigan, Minnesota, Mississippi, Missouri, Nebraska, Nevada, New Mexico, New York, North Dakota, Ohio, Oklahoma, Oregon, Pennsylvania, Rhode Island, South Carolina, South Dakota, Tennessee, Texas, Utah, Virginia, West Virginia, and Wisconsin.

^¶^ Number of respondents for some characteristics might not sum to overall total because of missing information; column percentages might not sum to 100% because of rounding. Reported totals are unweighted.

** Indicates the referent group used for t-tests to determine significant differences between levels of each characteristic for the prevalence of risk factors (p<0.05).

^††^ Indicates significant difference (p<0.05) based on t-tests in the prevalence of risk factors between the indicated level of each characteristic and the referent group.

^§§^ “Other race, non-Hispanic” includes respondents who reported they are of some other race group not listed in the survey question responses and are not of Hispanic origin.

^¶¶^ Includes general educational development certificate.

*** Includes self-employed persons.

^†††^ Includes students and homemakers.

**FIGURE:**
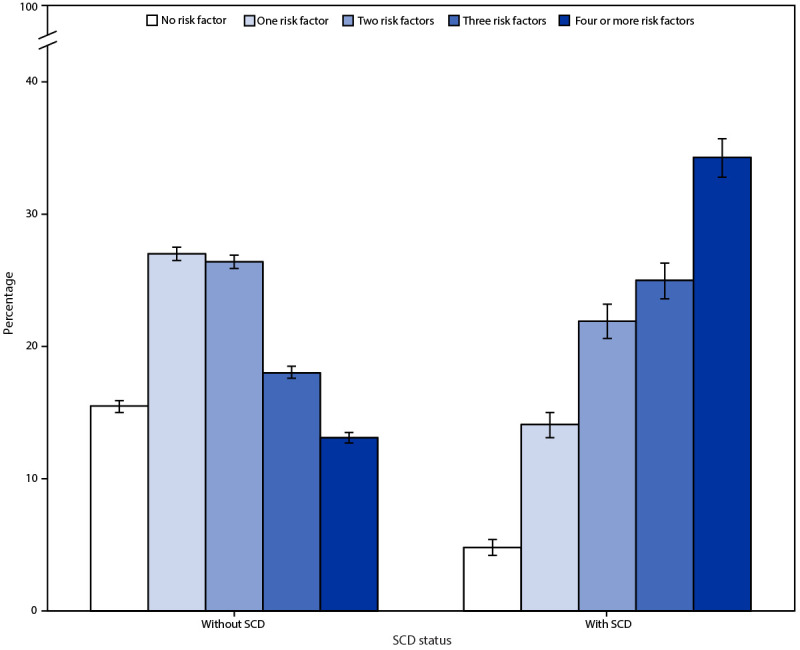
Proportion of adults aged ≥45 years with total number of risk factors,* by subjective cognitive decline status^†^ — Behavioral Risk Factor Surveillance System, United States,^§^ 2019 **Abbreviation:** SCD = subjective cognitive decline. * Total number of risk factors was defined as the sum of any of the following risk factors reported by the respondent: high blood pressure, not meeting the aerobic physical activity guideline, obesity, diabetes, depression, current cigarette smoking, hearing loss, or binge drinking. ^†^ SCD was defined as the self-reported experience of worsening confusion or memory loss in the previous year. ^§^ The following U.S. jurisdictions administered the SCD module in 2019: Alabama, Connecticut, District of Columbia, Florida, Georgia, Indiana, Iowa, Kansas, Louisiana, Maryland, Michigan, Minnesota, Mississippi, Missouri, Nebraska, Nevada, New Mexico, New York, North Dakota, Ohio, Oklahoma, Oregon, Pennsylvania, Rhode Island, South Carolina, South Dakota, Tennessee, Texas, Utah, Virginia, West Virginia, and Wisconsin.

Adults with each modifiable risk factor, except for binge drinking, were more likely to report SCD than were those without the risk factor (Table 2). Prevalence of SCD ranged from a high of 28.5% among persons with depression and 24.7% among those with hearing loss to 11.3% among those who reported binge drinking. SCD prevalence increased from 3.9% among those with no risk factors to 25.0% among those with four or more risk factors.

**TABLE 2:** Prevalence of subjective cognitive decline* among adults aged ≥45 years, by risk factor status^†^ and total number of risk factors^§^ — Behavioral Risk Factor Surveillance System, United States,^¶^ 2019

Risk factor	% (95% CI)	p-value **
**Overall**	**11.3 (11.0–11.6)**	**NA**
**High blood pressure**		
No	8.8 (8.4–9.3)	Ref
Yes	13.8 (13.3–14.3)	<0.001
**Not meeting aerobic physical activity guideline**		
No	8.3 (7.9–8.7)	Ref
Yes	14.5 (14.0–15.1)	<0.001
**Obesity**		
No	10.8 (10.3–11.2)	Ref
Yes	12.7 (12.1–13.3)	<0.001
**Diabetes**		
No	9.9 (9.6–10.2)	Ref
Yes	17.4 (16.5–18.4)	<0.001
**Depression**		
No	7.5 (7.2–7.8)	Ref
Yes	28.5 (27.4–29.6)	<0.001
**Current cigarette smoking**		
No	10.1 (9.7–10.4)	Ref
Yes	18.4 (17.3–19.6)	<0.001
**Hearing loss**		
No	9.7 (9.4–10.0)	Ref
Yes	24.7 (23.5–26.0)	<0.001
**Binge drinking**		
No	11.2 (10.9–11.6)	Ref
Yes	11.3 (10.2–12.4)	0.9
**Total no. of risk factors^§^**		
None	3.9 (3.4–4.4)	Ref
One	6.2 (5.8–6.7)	<0.001
Two	9.6 (9.0–10.2)	<0.001
Three	15.0 (14.2–15.9)	<0.001
Four or more	25.0 (23.9–26.2)	<0.001

**Abbreviations:** NA = not applicable; Ref = referent group; SCD = subjective cognitive decline.

* SCD was defined as the self-reported experience of worsening confusion or memory loss in the previous year.

^†^ Behavioral Risk Factor Surveillance System survey questions and calculated variables for 2019 are available at https://www.cdc.gov/brfss/questionnaires/pdf-ques/2019-BRFSS-Questionnaire-508.pdf and https://www.cdc.gov/brfss/annual_data/2019/pdf/2019-calculated-variables-version4-508.pdf, respectively. Not meeting the aerobic physical activity guideline was defined as answering “no” to question C11.01 or reporting <150 minutes per week of moderate-intensity aerobic activity, or <75 minutes per week of vigorous-intensity aerobic activity, or an equivalent combination of the two based on questions C11.02–C11.07 consistent with the Physical Activity Guidelines for Americans, 2nd edition (https://health.gov/our-work/nutrition-physical-activity/physical-activity-guidelines/current-guidelines). Obesity was defined as having a calculated body mass index of ≥30 kg/m^2^ based on self-reported height and weight from questions C08.17 and C08.18. High blood pressure, diabetes, depression, and hearing loss were defined as answering “yes” to questions C04.01 (excluding pregnancy-related high blood pressure), C06.11 (excluding pregnancy-related diabetes), C06.09, and C08.20, respectively. Binge drinking was defined as reporting having had one or more alcoholic beverages in the previous 30 days and responding “one or more” when asked how many times during the past 30 days they had had X [X = 5 for men and X = 4 for women] or more drinks on an occasion (questions C10.01 and C10.03, respectively). Current cigarette smoking was defined as reporting having smoked ≥100 cigarettes in their lifetime and now smoking every day or some days in response to questions C09.01 and C09.02, respectively.

^§^ Total number of risk factors was defined as the sum of any of the following risk factors reported by the respondent: high blood pressure, not meeting the aerobic physical activity guideline, obesity, diabetes, depression, current cigarette smoking, hearing loss, or binge drinking.

^¶^ The following U.S. jurisdictions administered the SCD module in 2019: Alabama, Connecticut, District of Columbia, Florida, Georgia, Indiana, Iowa, Kansas, Louisiana, Maryland, Michigan, Minnesota, Mississippi, Missouri, Nebraska, Nevada, New Mexico, New York, North Dakota, Ohio, Oklahoma, Oregon, Pennsylvania, Rhode Island, South Carolina, South Dakota, Tennessee, Texas, Utah, Virginia, West Virginia, and Wisconsin.

** p-value using t-tests for comparisons of prevalence of SCD between the following groups: 1) adults with versus without each risk factor and 2) adults with no risk factors versus one, two, three, or four or more risk factors.

## Discussion

In 2019, among adults aged ≥45 years in 31 participating states and DC, the most common potentially modifiable risk factors for ADRD were high blood pressure and not meeting the aerobic physical activity guideline; each was found in nearly one half of adults. Disparities in the prevalence of risk factors were observed by selected demographic characteristics, including race and ethnicity. Adults with SCD were more likely to report having modifiable risk factors (except binge drinking) and were more likely to report a higher number of risk factors than were those without SCD. Prevalence of SCD was highest among persons with depression, with hearing loss, and with four or more risk factors.

Consistent with previous reports (*1*,*3*), these findings indicate the prevalence of several modifiable risk factors was higher among American Indian or Alaska Native, Black or African American, and Hispanic populations than among other races and ethnicities. These findings are consistent with known understandings of chronic disease disparities which are influenced by differences in the social determinants of health.*** In combination with known racial and ethnic differences in the prevalence of ADRD, these findings help identify opportunities to improve health equity through prioritizing and tailoring public health strategies for those at highest risk (*1*,*4*–*6*). For example, CDC’s National Healthy Brain Initiative^†††^ supports culturally tailored interventions that address ADRD risk factors specifically for American Indian or Alaska Native, Black or African American, or Hispanic populations (*7*).

This analysis focused on SCD, an early indicator of possible future ADRD (*4*) and observed that adults with SCD were more likely to report almost all assessed risk factors, as well as a larger number of risk factors, than were those without SCD. The possible mechanisms of protection from dementia in relation to addressing modifiable risk factors are complex (*2*); however, early detection of SCD and associated risk factors might facilitate early intervention to slow the progression of ADRD and its symptoms. The earlier dementia is diagnosed, the sooner care can be provided, including building a care team, participating in support services and counseling, addressing other chronic conditions, and better managing medications (*8*). Future research might also seek to understand the relationship between an increasing number of risk factors and related risk for ADRD and evaluate multicomponent strategies or interventions that simultaneously address multiple risk factors.

The findings in this report are subject to at least six limitations. First, causality between risk factors and SCD cannot be inferred from a cross-sectional study, and not everyone who reports SCD will develop ADRD (*9*). Second, self-reported data might be subject to several biases, including recall and social desirability. Third, the low response rates could have resulted in response bias. Fourth, respondents with missing risk factor data were not excluded when calculating the total number of risk factors reported; however, findings were similar in a sensitivity analysis conducted excluding any missing values. Fifth, because data are from 31 states and DC, the findings of this report might not be nationally generalizable. Finally, although this analysis examined common modifiable risk factors for ADRD with available data in the 2019 BRFSS, they are only a subset of suggested risk factors. Major strengths of this study include the large sample size and ability to examine many risk factors and SCD.

Important milestones have been achieved in advancing a public health approach to address risk factors for ADRD in the United States. In 2021, the National Plan was updated to include a new goal to reduce risk factors for ADRD (*3*). Given the prevalence of modifiable risk factors for ADRD and anticipated growth of the older adult population and those with ADRD (*1*,*5*,*10*), this new goal has the potential to benefit a large proportion of U.S. adults. The findings in this report highlight opportunities to accelerate action, particularly among specific populations at high risk. Many evidence-based activities that support healthy aging and prevention and control of various chronic conditions, such as managing hypertension and promoting physical activity, can also serve as potential strategies to achieve this goal. For example, in addition to helping patients discuss concerns about memory loss, health care professionals should also screen patients for modifiable risk factors, counsel patients with risk factors, and refer them to effective programs and interventions where recommended. Public health professionals can implement policy, systems, and environmental strategies to address modifiable risk factors at the population level. Additional resources are available from the Building Our Largest Dementia Infrastructure Public Health Center of Excellence on Dementia Risk Reduction.^§§§^

**Summary**

**What is already known about this topic?**

The 2021 National Plan to Address Alzheimer's Disease (National Plan) included a goal to reduce the risk for Alzheimer disease and related dementias (ADRD).

**What is added by this report?**

Adults aged ≥45 years with subjective cognitive decline (SCD) were more likely to report four or more risk factors compared with those without SCD (34.3% versus 13.1%). Prevalence of SCD increased from 3.9% among adults with no risk factors to 25.0% among those with four or more risk factors.

**What are the implications for public health practice?**

Implementing evidence-based strategies that address modifiable risk factors can help achieve the National Plan’s goal to reduce risk for ADRD while promoting healthy aging.

## Footnotes

* Although the term “Alzheimer’s disease” is frequently used, this report uses “Alzheimer disease” in accordance with the American Medical Association Manual of Style 11th Edition and *MMWR* style.

† The following U.S. jurisdictions administered the SCD module in 2019: Alabama, Connecticut, District of Columbia, Florida, Georgia, Indiana, Iowa, Kansas, Louisiana, Maryland, Michigan, Minnesota, Mississippi, Missouri, Nebraska, Nevada, New Mexico, New York, North Dakota, Ohio, Oklahoma, Oregon, Pennsylvania, Rhode Island, South Carolina, South Dakota, Tennessee, Texas, Utah, Virginia, West Virginia, and Wisconsin.

§ SCD was defined as the self-reported experience of worsening confusion or memory loss in the previous year.

¶
https://www.uspreventiveservicestaskforce.org/uspstf/

**
https://www.thecommunityguide.org

††
https://www.cdc.gov/brfss/annual_data/2019/pdf/2019-response-rates-table-508.pdf

§§ BRFSS survey questions and calculated variables for 2019 are available at https://www.cdc.gov/brfss/questionnaires/pdf-ques/2019-BRFSS-Questionnaire-508.pdf and https://www.cdc.gov/brfss/annual_data/2019/pdf/2019-calculated-variables-version4-508.pdf, respectively. Not meeting the aerobic physical activity guideline was defined as answering “no” to question C11.01 or reporting <150 minutes per week of moderate-intensity aerobic activity, or <75 minutes per week of vigorous-intensity aerobic activity, or an equivalent combination of the two based on questions C11.02–C11.07 consistent with the Physical Activity Guidelines for Americans, 2nd edition (https://health.gov/our-work/nutrition-physical-activity/physical-activity-guidelines/current-guidelines). Obesity was defined as having a calculated body mass index of ≥30 kg/m^2^ based on self-reported height and weight from questions C08.17 and C08.18. High blood pressure, diabetes, depression, and hearing loss were defined as answering “yes” to questions C04.01 (excluding pregnancy-related high blood pressure), C06.11 (excluding pregnancy-related diabetes), C06.09, and C08.20, respectively. Binge drinking was defined as reporting having had one or more alcoholic beverages in the previous 30 days and responding “one or more” when asked how many times during the past 30 days they had had X [X = 5 for men and X = 4 for women] or more drinks on an occasion (questions C10.01 and C10.03, respectively). Current cigarette smoking was defined as reporting having smoked ≥100 cigarettes in their lifetime and now smoking every day or some days in response to questions C09.01 and C09.02, respectively.

¶¶ 45 C.F.R. part 46.102(l)(2), 21 C.F.R. part 56; 42 U.S.C. Sect. 241(d); 5 U.S.C. Sect. 552a; 44 U.S.C. Sect. 3501 et seq.

***
https://www.cdc.gov/chronicdisease/programs-impact/sdoh.htm

†††
https://www.cdc.gov/aging/funding/hbi/index.html

§§§
https://www.alz.org/professionals/public-health/public-health-approach/alz-association-efforts
